# Ionic Strength Investigation on the Interaction Between miR-155 and a PNA-Based Probe by Atomic Force Spectroscopy

**DOI:** 10.3390/biom15050634

**Published:** 2025-04-28

**Authors:** Davide Atzei, Francesco Lavecchia di Tocco, Anna Rita Bizzarri

**Affiliations:** 1 Biophysics and Nanoscience Centre, Department of Ecology and Biology (DEB), Università della Tuscia, Largo dell’Università, 01100 Viterbo, Italy; davide.atzei@unitus.it (D.A.); francesco.ditocco@unitus.it (F.L.d.T.); 2 Department of Biomedical Sciences and Technologies, Università Roma Tre, Viale Guglielmo Marconi, 00144 Rome, Italy

**Keywords:** microRNA, miR-155, peptide nucleic acids, atomic force spectroscopy, ionic strength

## Abstract

Peptide nucleic acids (PNAs) are synthetic analogues of DNA/RNA characterized by the absence of negative phosphate groups, which confer a low sensitivity to ionic strength for hybridization with respect to the canonical counterpart. PNAs are a suitable probe for miRNAs, as well as endogenous molecules of single-strand non-coding RNA whose dysregulation is often linked to several diseases. The interaction forces between PNA and microRNA-155 (miR-155), a multifunctional microRNA overexpressed in a variety of tumors, were investigated by Atomic Force Spectroscopy (AFS) in fluid under different conditions. We found that the unbinding forces acquired at the ionic strength of 150 mM for a rather wide range of loading rates (ΔF/Δt) can be described using the Bell–Evans model. This allows us to extract information on the kinetics and thermodynamic properties of the miR-155/PNA duplex. Additionally, we probed the unbinding forces and the target recognition times between miR-155 and PNA in the 50–300 mM ionic strength range. Our results indicate that both of these parameters are practically independent from the ionic strength in the analyzed range. The results provide information that is useful for a wider use of PNA in biosensors for diagnostics and therapeutics, even in situ.

## 1. Introduction

Peptide nucleic acids (PNAs) are synthetic analogues of DNA/RNA in which the negative sugar–phosphate backbones are replaced by unit repeats of 2-aminoethyl-glycine linked by a peptide bond, while the canonical nucleobases are connected to the backbone by a methylene bridge [1,2]. These chemical features confer some peculiar properties to PNA molecules, which can be exploited for various applications [3,4,5]. The peptide-like structure allows PNA molecules to escape from the activity of cellular nucleases or proteases, providing relatively high stability in cells or fluids, which is useful to reach specific targets, or for drug delivery [6,7]. Furthermore, the absence of negative phosphate groups makes PNA much less sensitive to ionic strength for hybridization with a complementary DNA/RNA strand in comparison to canonical (nucleic acid) counterparts [8]. Specifically, PNA can form a duplex with a DNA/RNA partner, and a high binding affinity, thermal stability, enzymatic resistance, and excellent mismatch discrimination [9]. Since hybridization between DNA/RNA partners requires a rather high ionic strength to screen repulsion between the negative charged partners, PNAs represent an extremely promising alternative for hybridization of a DNA/RNA target when a low ionic strength is required [10].

Recently, PNA molecules have been used as a hybridization partner for microRNAs (miRNAs), which are endogenous molecules of single-strand non-coding RNA, approximately 21–25 nucleotides in length. miRNAs play a fundamental role in regulating gene expression at the post-transcriptional level [11,12], and their dysregulation is often linked to the emergence of several disease states [13,14], including various types of cancer [15,16]. Several approaches have been developed and applied for miRNA detection; many of them require amplification and/or labelling, with high costs and long-term analysis [17].

Among techniques without amplification and labeling, PNA represents a promising alternative to complementary strands as a capture element. Bio Field Effect Transistor (BioFET) and electrochemical Impedance Spectroscopy (EIS) biosensors [18] reveal charges from the captured target within a distance from the electrode surface (called Debye length), in which the complementary strands are immobilized [19,20]. Since the Debye length progressively decreases as far as a higher ionic strength is applied [21], the use of PNA, combined with a low ionic strength, constitutes an effective strategy to enhance the detection level [22]. Nanopore Force Spectroscopy, which produces a peculiar electrical signature of the miRNA/probe duplex trapped in the pore that is drastically different than those detected for free miRNA or the probe [23], and could also take advantage of the use of PNA. Furthermore, PNA also represents a suitable probe in nanomechanical sensors, exploiting the force for discriminating against different targets [24].

Although PNA has been used in some applications, the interaction properties between miRNA and PNA and their interplay with the ionic strength are still not fully clarified. With the aim of investigating the interaction between miRNA and PNA, we have applied Atomic Force Spectroscopy (AFS), a nanotechnological tool which exploits the capabilities of the Atomic Force Microscopy (AFM) equipment, to monitor the inter- and intra-molecular forces of a single biomolecular system, well-complementing the results from bulk techniques [25]. AFS offers key advantages by directly measuring the interaction forces between the partners and providing detailed insights into kinetic and thermodynamic properties. Compared to Nanopore Force Spectroscopy, AFS enables direct observation of molecular interactions on a support, a feature that is particularly useful for biosensors, as it provides a more comprehensive understanding of molecular forces, including stability and dissociation rates [26].

Here, we have focused our attention on miR-155, a multifunctional miRNA that controls B cell growth, differentiation, and other processes, overexpressed or mutated in a variety of malignant tumor cells, including breast, colon, and hepatocellular carcinoma [27,28]. miR-155 is a good biomarker for a variety of malignancies and other illnesses, and its identification warrants significant attention in terms of prognosis and diagnosis [29,30,31]. Recently, we used PNA as a probe for the detection of miR-155 in a BioFET setup using an ionic strength at 150 mM to match physiological conditions by reaching a detection level of miR-155 down to 5 nM, combined with high specificity [32], higher than that obtained using a complementary strand in combination with a short polymer [33].

We have probed the interaction forces between miR-155 tethered to the tip of the AFM cantilever, and its complementary strand, PNAs, covalently anchored to a substrate, provided by the same gold-coated electrode used in the previous bioFET experiments [32]. The tip has approached the substrate to promote the formation of a duplex between the partners, while the subsequent retraction of the tip from the substrate yields the unbinding of the eventually formed complex by allowing us to extract the unbinding force.

First, we have investigated the unbinding forces of the duplex at the ionic strength of 150 mM by varying the loading rate (given by the variation in time of the applied force, ΔF/Δt) in a rather wide range. An analysis in the framework of the Bell–Evans model has revealed that the duplex is stable throughout the entire loading rate range by allowing us to extract information about its kinetics and thermodynamic properties. We have also investigated the unbinding force between miR-155 and PNA at a fixed loading rate, by varying the ionic strength in the 50–300 mM interval, which deserves some attention from the physiological point of view. Such an analysis has provided evidence for the fact that the interaction forces do not change substantially in the analyzed range of ionic strength. Additionally, we have evaluated the target recognition time, given by the time that the two partners spent in contact before the unbinding process, as a function of the ionic strength. We found that the target recognition time is characterized by a high variability; however, this trend is consistent with substantial independence on the ionic strength.

These results provide information that is useful for aware use of PNA as a probe in biosensing and nano-sensing of miRNAs whose detection is progressively becoming more important in diagnostics and therapeutics [34,35,36]. Moreover, the ability to work at a rather low ionic strength without substantial changes in the interaction with the target opens new possibilities for disease treatments, enhancing the effectiveness of therapies based on the inhibition or activation of specific microRNAs by PNA molecules [37,38].

## 2. Materials and Methods

### 2.1. Oligonucleotides

Single-stranded RNA oligonucleotides with the sequence of human miR-155-5p (5′-uaa ugc uaa ucg uga uag ggg-3′), called miR-155, were purchased from Metabion (Planegg, Germany). The producer purified the oligonucleotides by high-performance liquid chromatography–mass spectrometry (HPLC−MS). These oligonucleotides were resuspended in sterile 10 mM sodium phosphate buffer (NaPi, 8.1 mM Na_2_HPO_4_, 1.9 mM NaH_2_PO_4_, pH 7.8) and stored at 253 K. Work surfaces and equipment were decontaminated using RNaseZap (Ambion, Sigma-Aldrich Co.) St. Lous, MO, USA. The thiolated PNA oligomer with a complementary sequence to miR-155 was synthesized by biomers.net (Ulm, Germany) and shipped in dry form. The pellets were resuspended in sterile 10 mM sodium phosphate buffer (NaPi, 8.1 mM Na_2_HPO_4_, 1.9 mM NaH_2_PO_4_) in aliquots with a 100 µM concentration, miRs at pH = 7.8, and the PNA at pH = 6, and then stored at 253 K. Furthermore, 6-mercapto-1-Hexanol (MCH), and the other chemical reagents, were purchased from Sigma-Aldrich Co., Merck KgaA, Darmstadt, Germany.

### 2.2. Tips and Substrate Functionalization

For the AFS experiments, silicon nitride AFM tips with a nominal spring constant, k_nom_, of 0.06 N/m and of 0.3 N/m (cantilever B, MSNL-10; Bruker Corporation, Billerica, Massachusetts, USA) were used. The tips were functionalized with miR-155 using a flexible linker, N-hydroxysuccinimide-polyethyleneglycol-maleimide (NHS-PEG-MAL, 3.4 kDa, hereafter PEG) (Iris Biotech, Marktredwitz, Germany), by following the same procedure reported in ref. [39,40]. Briefly, the tips were cleaned in acetone (Sigma-Aldrich Co.) and UV-irradiated for 30 min to expose hydroxyl groups [41]. They were therefore incubated for 2 h at room temperature with a solution of 2% (volume/volume) 2-aminopropyl-triethoxysilane (APTES) (Acros Organics, Geel, Belgium) in chloroform (Sigma-Aldrich Co.), extensively washed with chloroform, and dried with nitrogen. The silanized tips were then immersed in a 1 mM solution of PEG in dimethylsulfoxide (DMSO) (Sigma-Aldrich Co.) for 3 h, allowing the NHS-ester groups of the PEG to bind to the amino groups of APTES. After washing with DMSO and microfiltered bidistilled water, the tips were incubated overnight at 277 K with 10 µL of 10 µM miR-155-SH in working buffer of pH 7.8, enabling the MAL groups of the PEG to react with the thiol group of miR-155-SH. Unreacted groups were passivated by incubation for 30 min with 1M ethanolamine hydrochloride, pH 8.5 (GE Healthcare, Chicago, IL, USA). The work surface and equipment were decontaminated using RNaseZap (Ambion (Austin, TX, USA); Sigma Aldrich Co. (St. Louis, MO, USA)). The gold-coated electrode surfaces (DRP-220AT-U75, with a gold sensing track area of 2.0 mm^2^ purchased from METROHM Italiana Srl, Origgio, Italy) were UV-irradiated for 30 min while immersed in hydrogen peroxide [41]. After washing with Milli-Q and drying with nitrogen, they were incubated for 4 h at 298 K with 13 µL of a solution of 5 µM PNAs in working buffer, pH 7.8, and subsequently passivated with 13 µL of a solution of 1 mM 6-mercapto-1-hexanol (MCH, Sigma-Aldrich Co.). All samples were stored in working buffer at 277 K.

### 2.3. AFS Experiments

AFS measurements were performed at room temperature with the Nanoscope IIIa/Multimode AFM (Veeco Instruments, Plainview, NY, USA) in working buffer at pH 7.8. The force, F, was evaluated by multiplying the cantilever deflection by its effective spring constant, determined according to the procedure in ref. [42]. Force curves were collected by approaching and retracting the cantilever, whose tip is functionalized with miR-155, towards the gold-coated surface of the sensor, in which PNA molecules were immobilized. The approaching phase was stopped upon reaching a preset maximum force value of 0.6 nN. The approach velocity was fixed at 50 nm/s, while the retraction velocity varied from 50 to 4200 nm/s. This led to different loading rates (LRs), given by the product of the cantilever retraction velocity (v) and the spring constant of the entire system (k_syst_), which was determined according to the procedure in ref. [43]. At each loading rate, more than thousands of force curves were acquired to guarantee the information had statistical significance. Curves characterized by a nonlinear trend before the jump-off were preliminary selected to extract those exhibiting the peculiar stretching features of the PEG linker, according to the procedure reported in [44]. Briefly, the nonlinear trend should be described by the worm-like chain (WLC) model, with a persistence length consistent with that of the used PEG (0.36 nm). Such a procedure helps to rule out multiple unbinding events. Control experiments (blocking) were performed by incubating the PNA-functionalized electrode surfaces with 50 µL of 5 µM miR-155 in working buffer for 90 min at 298 K, and by repeating the force curve acquisition over the same substrate.

## 3. Results and Discussion

**Figure 1:**
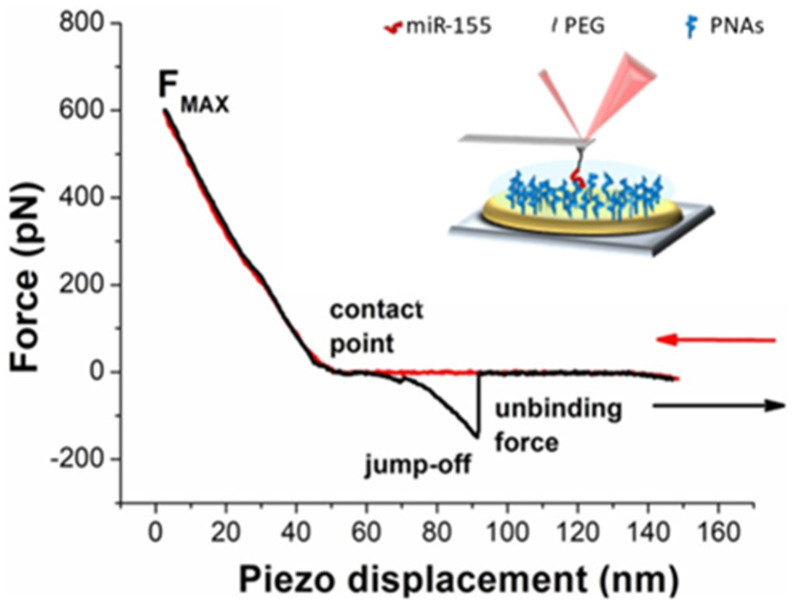
Representative AFS approach (red arrow) and retraction (black arrow) force curves, as a function of the piezo displacement, with miR-155 covalently attached to the tip through a PEG linker and PNA immobilized on the gold-coated substrate. Inset: Schematic representation of the experimental setup for AFS measurements.

The AFS experiments conducted to study the interaction between miR-155 and the PNA, at the single-molecule level, have been carried out by applying the same procedure followed for miR-155 and its complementary RNA strand [45]. Accordingly, force curves have been acquired by approaching the cantilever, whose tip was functionalized with miR-155, towards the substrate on which PNA molecules were immobilized, and then retracted from it. As the substrate, we have used the gold-coated surface of commercial electrodes, which are widely employed in biosensors [46,47], even in our group for detection miR-155, using antimir-155 as the probe [45]. Preliminarily, the active surface of commercial electrodes, before and after the various functionalization steps, has been analyzed by AFM imaging. The analysis, reported in the Supplementary Materials (see Figures S1 and S2 and Table S1), shows that the raw electrode surface is characterized by high roughness, consistent with the characteristics of the used electrodes. A decrease in roughness has been detected upon functionalizing the surface with the probe and MCH, providing evidence for the effectiveness of the functionalization procedure. A further decrease in the roughness has been observed upon adding the target (miR-155); this supports the capability of the probe immobilized on the substrate to bind the target. A PEG linker has been used for anchoring miR-155 to the tip to assure a higher mobility and then facilitating the biorecognition events. Furthermore, we remark that the use of PEG plays an important role in the discrimination between specific and nonspecific unbinding events [48]. AFS experiments were first conducted at the ionic strength of 150 mM as a function of the loading rate. More specifically, the approaching loading rate, R, was kept fixed at 21 nN/s, while the retraction one has been in the 5–1000 nN/s range. As commonly observed in AFS experiments, all of the approaching curves are characterized by almost the same trend as that shown in Figure 1 (red curve). Briefly, the curve takes on values around zero up to the contact point, at which point a physical contact between the tip and the substrate occurs. Beyond this point, the repulsive forces cause an upward linear deflection of the cantilever, stopped when the maximum applied force (F_max_) is reached. During this phase, the very few molecules on the tip (ideally a single molecule), and those on the substrate, may undergo a biorecognition process, eventually leading to a duplex formation. Retraction curves exhibit a rather large variability, depending on the kind of interaction between the tip and the substrate. When there is no interaction, the retraction curve practically follows the same trend of the approaching one. When a nonspecific (e.g., adhesion) or specific (i.e., duplex formation) interaction occurs, a jump-off event is recorded as far as the cantilever spring force overcomes the interacting forces [25,49]. More specifically, if a duplex is formed, a nonlinear trend, reflecting the characteristic nonlinear PEG stretching, is expected to be observed before the jump-off; the corresponding extension provides the unbinding force of the duplex.

A representative example of nonlinear stretching is shown in Figure 1 (black curve). Curves characterized by a nonlinear trend in the retraction phase have been further analyzed to select those that can be attributed to specific unbinding events, according to the procedure previously described (see also, Material and Methods) [44].

**Figure 2:**
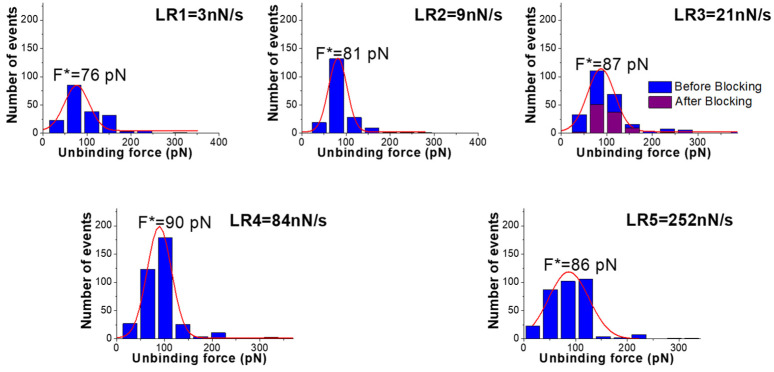
Histograms (blue columns) of the unbinding forces for the miR-155/PNA complex from AFS measurements carried out at different loading rates; the used cantilever has a nominal spring constant k of 0.06 N/m. The most probable unbinding force value (F*) has been determined from the maximum of the main peak of the histogram fitted by a Gaussian function (red curve). At the loading rate of 21 N/s, the histogram of the unbinding forces after blocking (purple columns) is also shown.

Figure 2 shows the histograms of the extracted unbinding forces (blue columns) recorded at five different loading rates, obtained with a cantilever that has a nominal spring constant k of 0.06 N/m. In all of the cases, the histograms are characterized by a single mode distribution, which can be well-described by a Gaussian curve (see red lines in Figure 2), whose peak gives the most probable unbinding force (F*). In all cases, the unbinding force values are characterized by a high spread, which can be attributed to different factors, such as molecular orientation heterogeneity, the existence of several conformations, possible multiple binding sites, etc. Indeed, such a variability is a common feature of the AFS data of biomolecular systems, and it is well-ascertained in the literature (see, e.g., ref. [25]). The values of the most probable unbinding forces, shown in Figure 2, follow a trend that is substantially consistent with a progressively increasing trend with the loading rates.

To further assess the specificity of the extracted unbinding events, a blocking experiment has been conducted by incubating the PNA-functionalized electrode surfaces with a solution containing miR-155, and then they are rinsed. The force curve acquisition has been repeated using the miR-155-functionalized tip and a loading rate of 21 nN/s. The resulting histogram, also shown in Figure 2 (purple columns), reveals a decrease in events of more than 50%. We also note that the histogram, after blocking, maintains almost the same shape, suggesting there is some residual activity upon blocking, similar to what has been observed in other experiments [40]. These results confirm the specificity of the interaction between miR-155 and PNA. AFS experiments have been also conducted by using a cantilever with a higher nominal spring constant (0.3 nN/s). Even for this set of curves, the most probable unbinding forces have been extracted from histograms; these data having been reported in the Supplementary Materials (see Figure S3).

**Figure 3:**
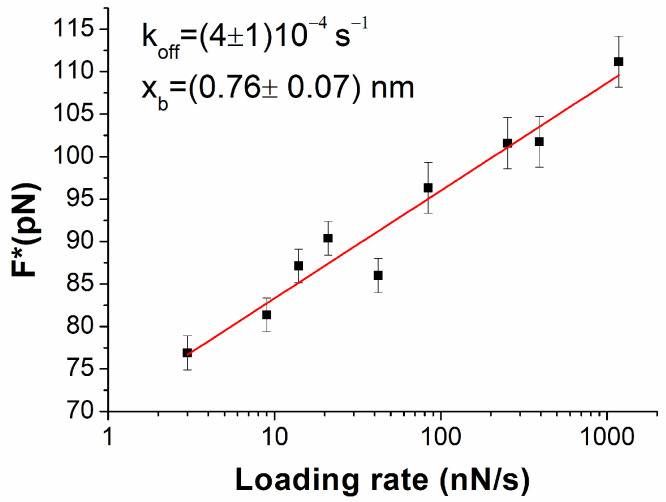
The most probable unbinding force, F*, vs. the logarithm of the loading rate for the miR-155/ PNAs duplex. Red continuous line is the best fit by the Bell−Evans model with equation 1; the extracted values for the k_off_ and x_β_ parameters are reported.

**Figure 4:**
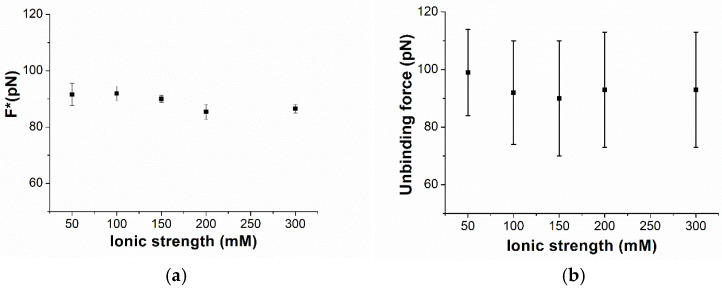
(**a**) The most probable unbinding forces F* and (**b**) the average unbinding forces for the miR-155/ PNAs duplex from AFS experiments conducted at the loading rate of 21 nN/s and at different ionic strengths.

The most probable unbinding forces have been plotted as a function of the logarithm of the loading rate in Figure 3. The forces globally follow a linear trend throughout the whole loading rate range. Such behavior agrees with the Bell–Evans model, which describes the unbinding process of a complex under the application of an external force in terms of a progressive decrease in the energy barrier between the bound and the unbound state of the complex [50,51]. In this framework, the dependence of the most probable unbinding forces on the loading rate can be described by the following equation [50,51]:

(1) F^∗=(k_B T)/x_βln (r x_β)/(k_off k_B T)

where k_B_ is the Boltzmann constant, T is the absolute temperature, k_off_ is the dissociation rate constant, and x_β_ is the width of the energy barrier along the direction of the applied force. A fit of the data in Figure 3 by Equation (1) has led us to determine that k_off_ = (4 ± 1) 10^−4^ s^−1^ and x_β_ = (0.76 ± 0.07) nm. The extracted k_off_, and the corresponding lifetime τ of the complex (τ = 1/k_off_ = 2.5·10^3^ s), indicate a rather stable interaction between miR-155 and PNA. Notably, the value of k_off_ is higher than that measured by SPR k_off_ = (3 ± 1) 10^−5^ s^−1^, an it is obtained using SPR gold sensor chips as the substrate. However, similar discrepancies between the AFS and SPR results have also been observed in other cases, and they could be due to the slightly different experimental conditions applied in the two techniques [52]. In our case, the use of electrodes instead of SPR sensor chips should also be considered. The evidence that the unbinding forces of the miR-155/PNA duplex follow a linear trend in the Bell–Evans plot in a rather wide range of loading rates indicates that, even under a rather high mechanical stress, the unbinding of the duplex can be described substantially by the same process. Since the increase in the loading rate is assumed to yield a progressive decrease in the energy barrier between the bound and unbound state, without substantially altering the mechanism of the unbinding process of the duplex, this can be put into a relationship to some robustness of the duplex with respect to mechanical stress. Such a finding provides a ground for the use of PNA as a probe for miRNAs, even in nano-sensors. To check the specificity of the interaction, we have repeated the experiment by using a tip functionalized with miR-141 against the substrate, in which PNA molecules that are complementary for miR-155 were anchored. The corresponding histogram of the unbinding forces in Figure 2 (purple columns) reveals a fraction of events with features consistent to specific events of less than 10%, with a drastic reduction with respect to what has been observed with miR-155. We then investigated the unbinding forces of the duplex by varying the ionic strength in the 50–300 mM range, with the loading rate being kept fixed at 21 nN/s. For each ionic strength, a thousand force curves have been acquired and analyzed. The unbinding force curves attributed to specific biorecognition events have been extracted and cast into a histogram (see Figure S4 in the Supplementary Materials). Similar to what has been observed for the ionic strength of 150 mM, the histograms at the different ionic strengths exhibit a single mode distribution. Figure 4a shows the most probable extracted force, with the error as evaluated by a fitting with a Gaussian curve as a function of the ionic strength. For all of the ionic strengths, the force assumes values around 90 pN, with rather small variability. The average values of the unbinding forces, shown in Figure 4b, are slightly higher with respect to those found for the most probable forces, however, with a much larger spread in agreement with what is usually observed for the unbinding force [25]. Furthermore, the spread is almost the same for all of the ionic strengths. These results indicate that the unbinding forces are almost the same at the various ionic strengths. From a general point of view, the evidence that the force required to break the duplex is independent of the ionic strength in the 50–300 mM range is consistent with the hypothesis that the ionic strength of the surrounding environment does not affect the interaction properties between the strands. This should be discussed in connection with the use of PNA as an alternative probe, with respect to the complementary strand, in electrochemical biosensors. As already mentioned, a high ionic strength is required to allow an effective binding of the complementary strand RNA/DNA probe with the target, while a high ionic strength reduces the region from the electrode where the charges can be detected. The fact that the interaction of the target with PNA is independent of the ionic strength removes the requirement of a high ionic strength and reduces the limitation due to the Debye length, allowing us to improve the detection capability of the electrochemical biosensor. Furthermore, independence on the ionic strength of the hybridization process deserves a particular relevance for the use of PNA as a probe in nano-sensors. Since the ionic strength could slightly change from site to site, the substantial independence of the interaction properties of PNA with the miRNA on the ionic strength supports reliability measurements of the nano-sensor.

**Figure 5:**
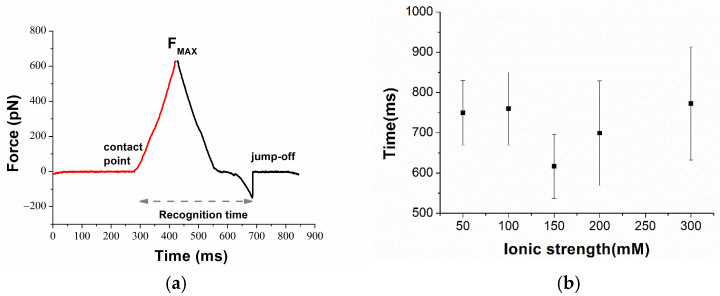
(**a**) Plot of the approaching and retraction force curves vs. time for force curves of miR-155/PNA duplex; the time attributed to the recognition time is indicated as a dashed gray arrow. (**b**) Average recognition times for the unbinding event of the miR-155/PNA duplex at different ionic strengths.

To complete our analysis, we have also estimated the time during which the partners are in contact during the acquisition of a force curve by measuring the time between the tip sample contact point up to the jump-off point along the time axis for the curves. We have followed the procedure developed in ref. [24] and depicted in Figure 5a, in which the approaching and retraction curves are successively plotted as a function of time. Such a time, also called target recognition time, gives the time that the two partners spent in contact, including the time requested to form the duplex, the time during which the duplex is formed, and, finally, the time before a complete unbinding occurs. Figure 5b shows the average of the target recognition time from force curves at the fixed loading rate of 21 nN/s, and at an ionic strength in the 50–300 mM range. The target recognition time falls in the 600 ms to 750 ms interval, with all of the data being characterized by a large spread. Because of the large variability, a definite trend of the target recognition time with the ionic strength cannot be extracted; however, the found values suggest that they are independent from the ionic strength.

In summary, these results indicate that the unbinding force, and likely the global time during which the miR-155 and PNA strand are in contact, are not affected by the ionic strength.

## 4. Conclusions

The interaction properties between miR-155, which is a suitable biomarker for several diseases, and the complementary PNA molecules, immobilized on commercial gold electrodes as the substrate, were investigated at different ionic strengths by AFS. Since PNA molecules have the capability to hybridize with RNA, even at a low ionic strength, they represent a very promising class of molecules to be used as probes in different biosensing setups. The immobilization of the PNA probe on the gold-coated surface allowed us to closely approach the conditions widely used in electrochemical biosensors. We found that the dissociation rate of the miR-155/PNA duplex is about 10^5^ s^−1^, at the ionic strength of 150 mM, which closely approaches the physiological condition. This result is indicative that miR-155 and PNA give rise to a rather stable complex, consistent with what was previously found by SPR using a different binding surface. The linear trend of the unbinding force of the duplex as a function of the logarithm loading rate, predicted by the Bell–Evans model, was found to be satisfied in a rather wide range of loading rates. This supports the robustness of the miR-155/PNA duplex with respect to mechanical stress, and it deserves some interest in force sensing. The unbinding force of the duplex analyzed, at a given loading rate, was found to be almost independent in the 50–300 mM ionic strength range. Such findings demonstrate the relevance of using PNA in electrochemical biosensors, giving the possibility to attenuate the Debye screening at the high ionic strength required for canonical nucleic acids hybridization. Along with the same direction, we found that the time recognition required—including the formation and the disruption of the duplex—is practically independent from the ionic range. This further supports the low sensitivity of the miR-155/PNA duplex interaction properties on the ionic strength. Globally, our results support the use of PNA for the biosensing and nano-sensing of miRNA in a wide range of ionic strengths, even for the in loco analysis, where the environmental conditions could slightly vary from site to site.

## Data Availability

Data are contained within the article.

## Supplementary Materials

The following supporting information can be downloaded at: https://www.mdpi.com/article/10.3390/biom15050634/s1, Figure S1: AFM. Figure S2: 2D AFM. Figure S3: Histograms (blue columns) of the unbinding forces for the miR-155/PNA complex from AFS measurements using a cantilever with a nominal spring constant, k = 0.3 N/m. Figure S4: Histograms (blue columns) of the release forces for the miR-155/PNA complex, performed at different ionic using a cantilever with a nominal elastic constant, k = 0.06 N/m. Table S1: Roughness analysis of electrodes at different functionalization steps. Reference [53] is cited in Supplementary Materials file.
