# Supplementary material for: Ionic Strength Investigation on the Interaction Between miR-155 and a PNA-Based Probe by Atomic Force Spectroscopy
Source: Biomolecules. 2025 Apr 28;15(5):634. doi: 10.3390/biom15050634 (PMC12109014; doi:10.3390/biom15050634)
Supplement: Supplementary file 1 [file biomolecules-15-00634-s001.zip › biomolecules-3570899-supplementary.pdf]

SUPPORTING MATERIALS

Ionic strength investigation on the interaction between miR-155 and a PNA-based probe by Atomic Force Spectroscopy

Davide Atzei[1], Francesco Lavecchia di Tocco[1,2] and Anna Rita Bizzarri[1, *]

[1]Biophysics and Nanoscience Centre, DEB, Università della Tuscia, Largo dell'Università, 01100 Viterbo, Italy

[2]Department of Biomedical Sciences and Technologies, Università Roma Tre, Viale Guglielmo Marconi, 00144 Rome, Italy.

Methods

Atomic Force Microscopy (AFM) measurements were performed at room temperature with the Nanoscope IIIa/Multimode AFM (Veeco Instruments, Plainview, NY, USA). All measurements were done in Tapping Mode AFM (TM-AFM), using 0.01 – 0.025 Ohm-cm Antimony (n) doped Si tip (NCHV-A; Bruker Corporation) with a nominal spring constant of 42 N/m, operating at a resonance frequency of about 320 kHz and at an amplitude set point to the 95% of the free amplitude value. TM-AFM images of 10 x 10μm^2 scan size were recorded. Roughness analysis of the AFM images was performed using the analysis tools of the Nanoscope software and the WSxM 4.0 [1].

AFM imaging of functionalized electrode surfaces

The gold-coated surface of commercial electrodes at the various functionalization steps, as described in Materials and Methods Section, has been preliminarily analyzed by AFM imaging [2]. Figure S1a shows representative 2D and 3D images of the raw substrate after cleaning. The surfaces are generally characterized by a marked roughness at different length scales with the presence of several small spots, with heights ranging from 5 nm to 60 nm, in agreement with the features of the electrodes. Representative images of the substrates functionalized with the probe (PNA) and MCH, before and after the addition of the target (miR-155) are shown in Figure S1b and Figure S1c, respectively. At a visual inspection, no significant variations with respect to raw surfaces emerge. With the aim of extracting some information about the possible effects as due to the functionalization procedures, a statistical analysis of the roughness has been carried out. First, we analyzed the roughness after flattening of 10x10 μm^2 images before, after functionalization and upon adding the target. The average and the corresponding standard deviation of the roughness, for a collection of 30 images, are

reported in Table S1 (column 2). We note that the roughness is characterized by a rather high variability and no significant differences among the samples are detected. Successively, we have restricted our analysis to a smaller region, by taking into consideration areas with approximate dimensions of 2x2 μm^2. Regions where sharp variations occur have been excluded by the analysis; examples having been given by the blue squares in Figure S1.

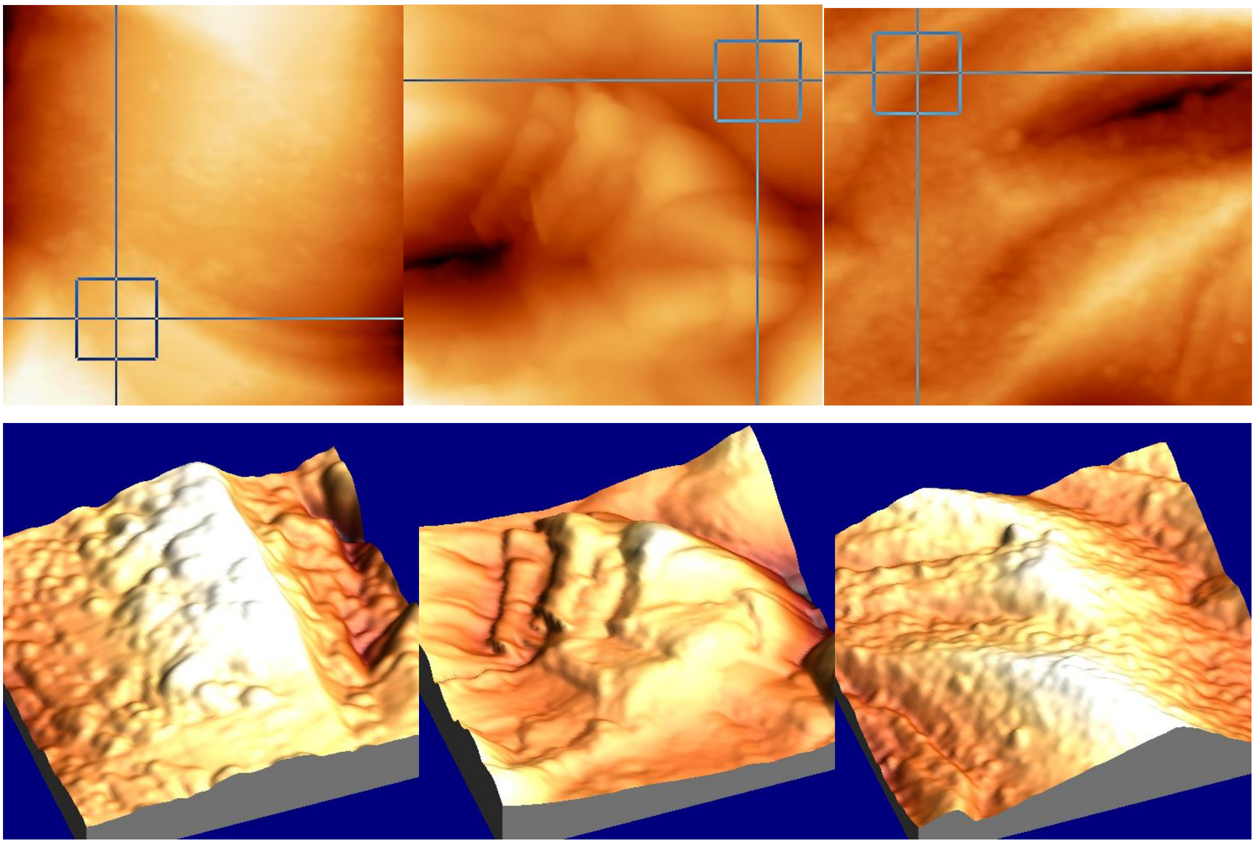

(a) (b) (c)

Figure S1. AFM images of: (a) raw electrodes; (b) electrodes functionalized with PNA and MCH; (c) electrodes functionalized with PNA and MCH upon the addition of miR-155

Results are also reported in Table S1 (column 3). We note that the roughness undergoes a marked decrease by passing from raw to functionalized surfaces, with a further decrease after the addition of the target (miR-155). These results generally indicate that the functionalization procedure yields some change in the coverage of the electrode surface without strongly affecting their topology at the analyzed ionic strength.

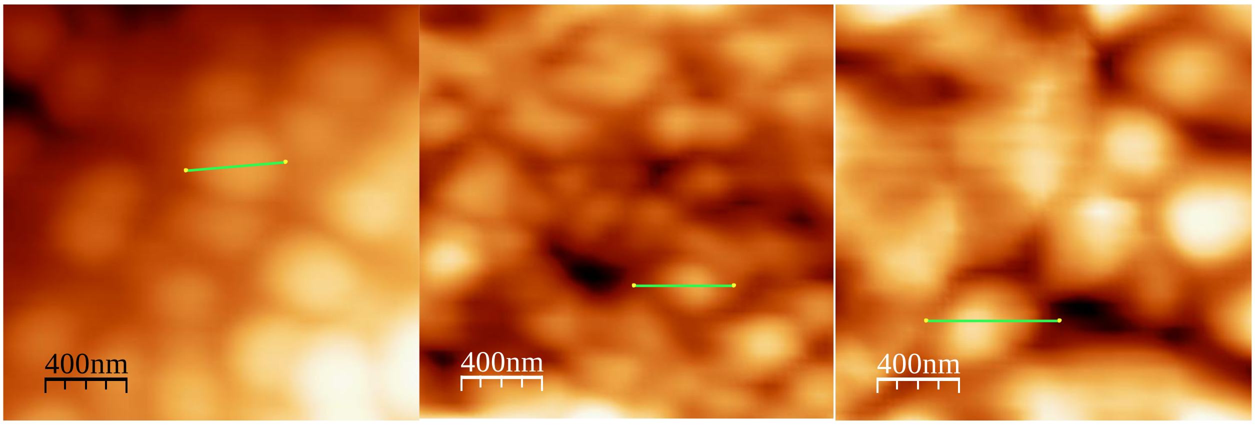

(a) (b) (c)

Figure S2. 2D AFM images in different zoomed-in areas of: (a) raw electrodes; (b) electrodes functionalized with PNA and MCH and (c) electrodes functionalized with PNA and MCH upon the addition of miR-155.

Table S1. Roughness analysis of electrodes at different functionalization steps. The roughness was calculated from ten regions for two different areas.

**SAMPLE**	**Roughness 10x10 nm**	**Roughness 2x2 nm**
Raw electrodes	53 ± 15	59 ± 6
Functionalized electrodes	68 ± 20	12 ± 2
Functionalized electrodes upon adding miR-155	50± 17	4.6 ± 0.7

k=0.3 N/m

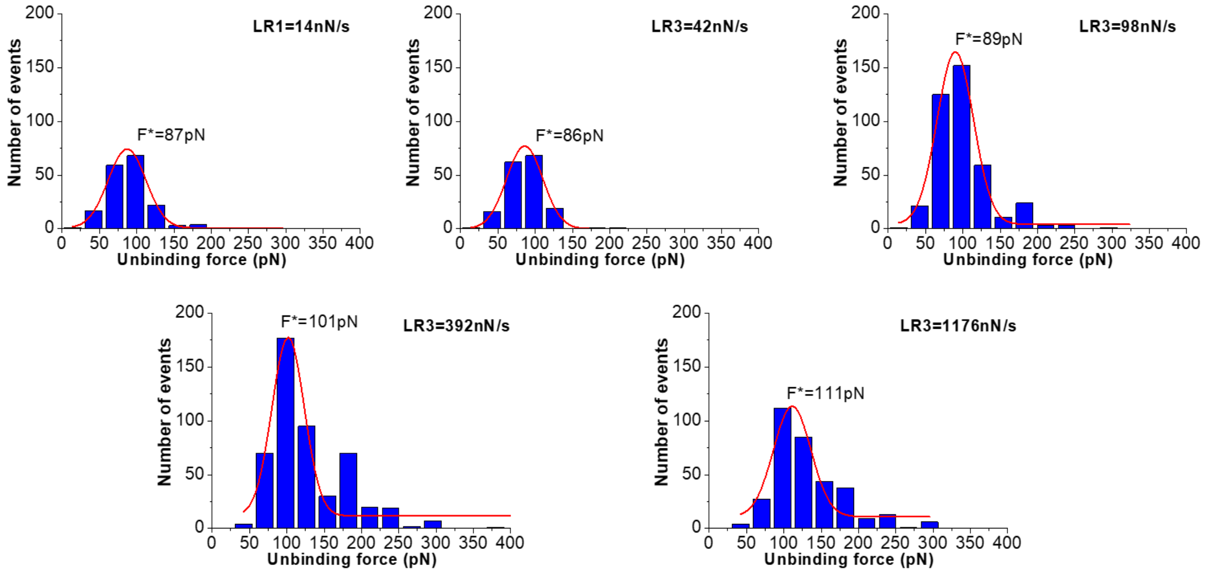

Figure S3. Histograms (blue columns) of the unbinding forces for the miR-155/PNA complex from AFS measurements carried out at increasing loading rates with cantilever with a nominal spring constant, k= 0.3N/m. The most probable unbinding force value (F*) has been determined from the maximum of the main peak of the histogram by fitting with a Gaussian function (red curve).

k=0.06 N/m

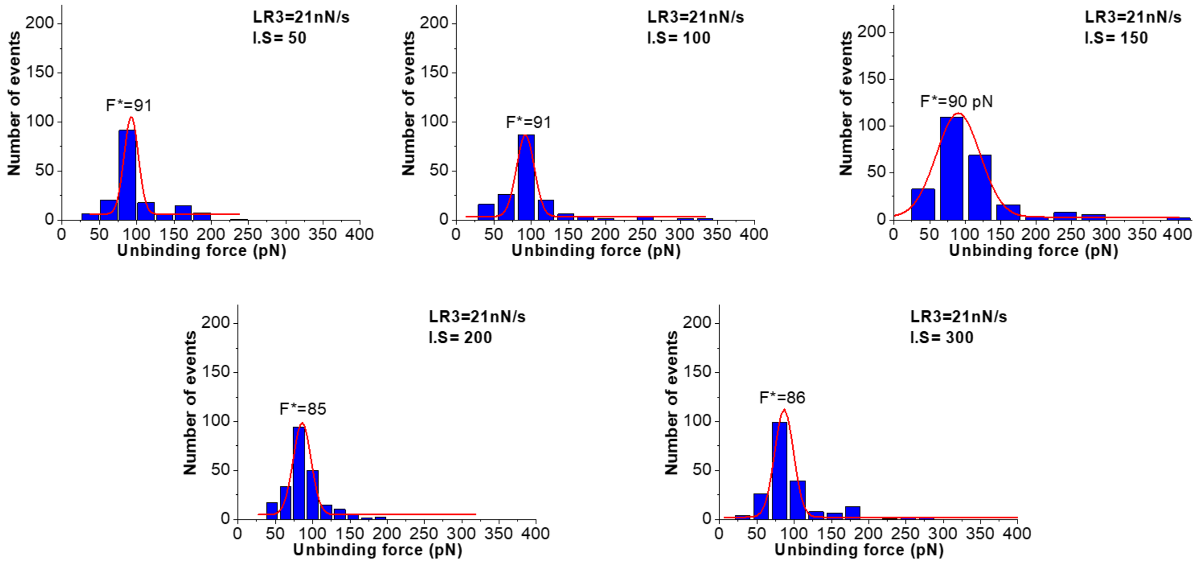

Figure S4. Histograms (blue columns) of the release forces for the miR-155/PNA complex, performed at different ionic strengths, derived from AFS measurements performed at a constant loading rate of 21 N/s with cantilever with a nominal elastic constant, k= 0.06 N/m. The most probable value of the release force (F*) was determined from the maximum of the main peak of the histogram by fitting it with a Gaussian function (red curve).

References

[1] I. Horcas, R. Fernández, J. M. Gómez-Rodríguez, J. Colchero, J. Gómez-Herrero, and A. M. Baro, "WSXM: a software for scanning probe microscopy and a tool for nanotechnology," *Rev Sci Instrum*, vol. 78, no. 1, 2007, doi: 10.1063/1.2432410.

[2] J. L. Hutter and J. Bechhoefer, "Calibration of atomic-force microscope tips," *Review of Scientific Instruments*, vol. 64, no. 7, pp. 1868–1873, 1993, doi: 10.1063/1.1143970.
